# Bioreactor‐manufactured cartilage grafts repair acute and chronic osteochondral defects in large animal studies

**DOI:** 10.1111/cpr.12653

**Published:** 2019-09-06

**Authors:** Andreja Vukasovic, Maria Adelaide Asnaghi, Petar Kostesic, Helen Quasnichka, Carmine Cozzolino, Maja Pusic, Lauren Hails, Nuala Trainor, Christian Krause, Elisa Figallo, Giuseppe Filardo, Elizaveta Kon, Anke Wixmerten, Drazen Maticic, Graziella Pellegrini, Wael Kafienah, Damir Hudetz, Tim Smith, Ivan Martin, Alan Ivkovic, David Wendt

**Affiliations:** ^1^ Department of Histology and Embriology, School of Medicine University of Zagreb Zagreb Croatia; ^2^ Department of Biomedicine University Hospital Basel, University of Basel Basel Switzerland; ^3^ Clinic for Surgery, Ophthalmology & Orthopaedics, Veterinary Faculty University of Zagreb Zagreb Croatia; ^4^ School of Cellular and Molecular Medicine University of Bristol Bristol UK; ^5^ Holostem Terapie Avanzate SRL Modena Italy; ^6^ Octane Biotech Kingston Ontario Canada; ^7^ PreSens Precision Sensing GmbH Regensburg Germany; ^8^ Fin‐Ceramica Faenza SPA Bologna Italy; ^9^ IRCCS Istituto Ortopedico Rizzoli Bologna Italy; ^10^ Department of Orthopaedic Surgery University Hospital “Sveti Duh,” Zagreb Croatia; ^11^ Department of Surgery University Hospital Basel, University of Basel Basel Switzerland; ^12^ Department of Biomedical Engineering University Hospital Basel, University of Basel Basel Switzerland; ^13^ Cellec Biotek AG Basel Switzerland; ^14^ Present address: General Hospital Bjelovar Bjelovar Croatia; ^15^ Present address: School of Biosciences Cardiff University Cardiff United Kingdom; ^16^ Present address: Humanitas University Department of Biomedical Sciences Milan Italy; ^17^ Present address: Humanitas Clinical and Research Center Milan Italy

**Keywords:** bioreactor, cartilage repair, large animal study, manufacturing, osteochondral, tissue engineering

## Abstract

**Objectives:**

Bioreactor‐based production systems have the potential to overcome limitations associated with conventional tissue engineering manufacturing methods, facilitating regulatory compliant and cost‐effective production of engineered grafts for widespread clinical use. In this work, we established a bioreactor‐based manufacturing system for the production of cartilage grafts.

**Materials & Methods:**

All bioprocesses, from cartilage biopsy digestion through the generation of engineered grafts, were performed in our bioreactor‐based manufacturing system. All bioreactor technologies and cartilage tissue engineering bioprocesses were transferred to an independent GMP facility, where engineered grafts were manufactured for two large animal studies.

**Results:**

The results of these studies demonstrate the safety and feasibility of the bioreactor‐based manufacturing approach. Moreover, grafts produced in the manufacturing system were first shown to accelerate the repair of acute osteochondral defects, compared to cell‐free scaffold implants. We then demonstrated that grafts produced in the system also facilitated faster repair in a more clinically relevant chronic defect model. Our data also suggested that bioreactor‐manufactured grafts may result in a more robust repair in the longer term.

**Conclusion:**

By demonstrating the safety and efficacy of bioreactor‐generated grafts in two large animal models, this work represents a pivotal step towards implementing the bioreactor‐based manufacturing system for the production of human cartilage grafts for clinical applications.

https://doi.org/10.1111/cpr.12625

## 1 INTRODUCTION

**Figure 1:**
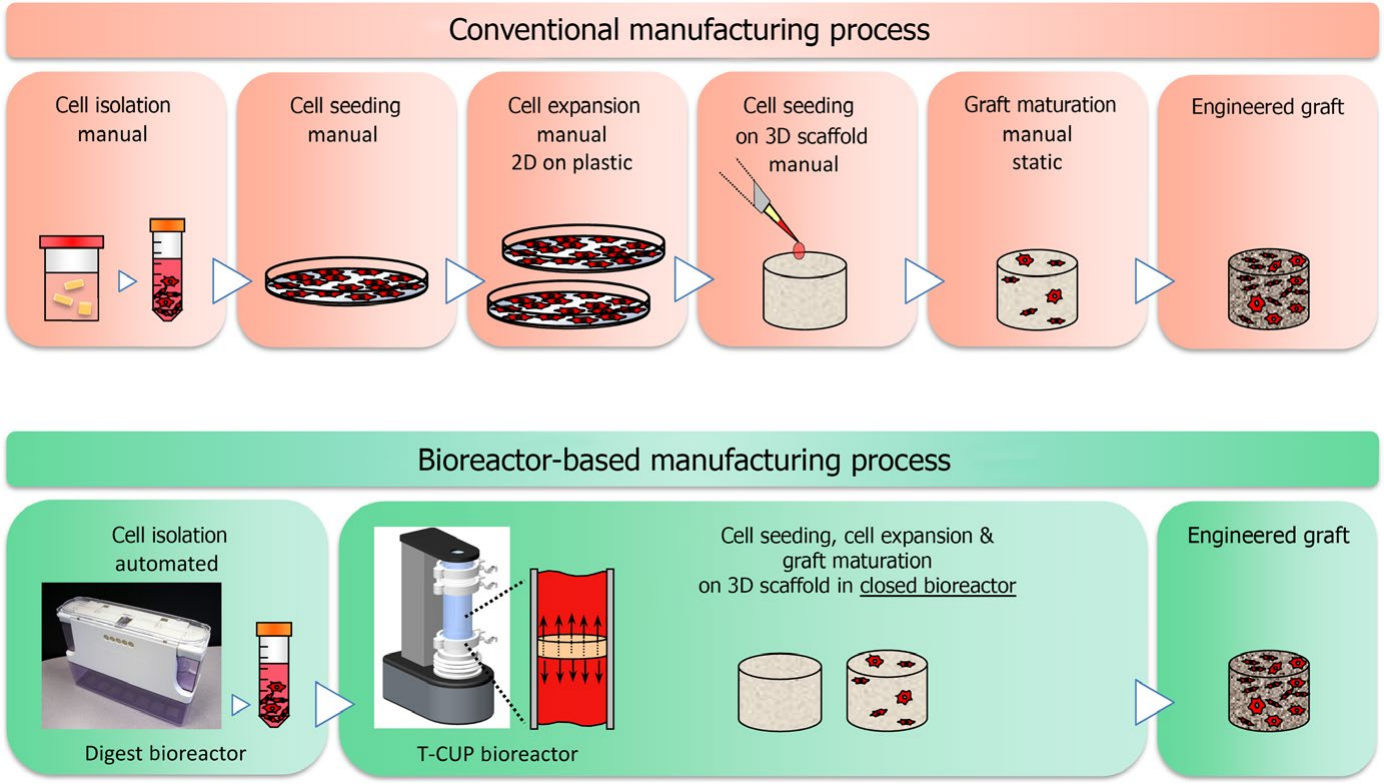
Conventional manufacturing processes used to produce the engineered grafts are based on traditional bench‐top manual culture methods. These manual procedures require a large number of labour‐intensive manipulations that pose challenges towards regulatory compliance and ultimately result in high manufacturing costs in the long term. As an alternative, bioreactor‐based production systems, which automate and control the bioprocesses, have the potential to overcome the limitations associated with conventional manufacturing methods, facilitating regulatory compliant and cost‐effective production of engineered cartilage grafts for widespread clinical use

In a recent phase I clinical trial, we aimed to treat articular cartilage defects with cartilage tissue grafts, which were engineered from nasal cartilage chondrocytes.^1^ Given the highly promising clinical data that we acquired in that study, we now aim to address critical manufacturing related issues, which could ultimately impede the translation of this therapy into widespread clinical use. Since the cartilage grafts in our study were produced by conventional manual tissue engineering methods, the production process was lengthy, labour‐intensive and would possess inherent variability among operators Figure 1. A manufacturing system based on these manual processes may be challenging to standardize, thus presenting obstacles towards regulatory compliance, and may ultimately incur high operating costs and challenges for upscaling, thus presenting significant barriers towards economic viability. Alternatively, automated bioreactor‐based manufacturing systems have the potential to overcome these limitations, breaking down regulatory and economic barriers, allowing engineered tissue therapies to reach their full clinical potential^2^ Figure 1.

We have previously demonstrated that bioreactor‐based perfusion of a cell suspension directly through the pores of a 3D scaffold enhances the cell seeding efficiency and cell distribution compared to conventional manual methods.^3^ We then demonstrated that culturing cell‐seeded constructs under perfusion supported the development of a viable and uniform tissue graft during prolonged culture.^4^ Chemo‐optic microsensors were also integrated into the bioreactor for continuous online measurements of oxygen levels in the perfused culture medium. With the prospect of clinical applications, we also upscaled the perfusion bioreactor system in order to engineer clinically relevant, large‐scale cartilage grafts suitable for treating lesions in a human knee.^5^ After establishing these fundamental building blocks for a bioreactor‐based production system, we then established an innovative and streamlined approach to engineer human cartilage grafts within a single bioreactor unit, from the introduction of primary chondrocytes freshly isolated from a biopsy, through the generation of a mature cartilaginous tissue graft.^6^

In this work, we have adapted and integrated our previously described bioreactor technologies and bioprocesses in order to establish an automated manufacturing platform for the production of nasal chondrocyte‐based engineered grafts. Cartilage grafts were manufactured in the bioreactor‐based system at a centralized GMP facility and were first assessed in a large animal study based on an acute osteochondral defect in sheep. Following the promising results from the acute defect study, we next assessed grafts generated in our manufacturing system in a more challenging and clinically relevant chronic defect sheep model. The results of this work represent a pivotal step towards implementing the bioreactor‐based manufacturing system for the production of human cartilage grafts for clinical applications.

## 2 MATERIAL AND METHODS

### 2.1 Scaffold preparation

The bilayered biomimetic osteochondral scaffold used in these studies (Fin‐Ceramica Faenza SpA) had a cylindrical shape, 25 mm in diameter and 5 mm in thickness for bioreactor‐generated cell‐based grafts and a 6 mm diameter for cell‐free scaffold implants. The top layer of the scaffold (3 mm in thickness) consisted of equine type I collagen, and the bottom layer (2 mm in thickness) was made of a mineralized blend of type I collagen and Mg‐HA, mimicking the structure and biochemistry of cartilage and subchondral bone.^7^, ^8^ The scaffold was developed through a bio‐inspired process, as previously described.^9^

### 2.2 Bioreactor‐based manufacturing

The bioreactor‐based manufacturing process is comprised of four main phases:

- *Cartilage tissue digestion phase*, in which the cartilage biopsy is enzymatically digested to liberate isolated chondrocytes in a cell suspension.
- *Cell seeding phase*, in which isolated primary chondrocytes are efficiently and uniformly seeded throughout the volume of the scaffold.
- *3D proliferation phase*, in which chondrocytes are extensively expanded in number to colonize the volume of the scaffold.
- *Differentiation phase*, in which the 3D expanded chondrocytes are re‐differentiated.

**Figure 2:**
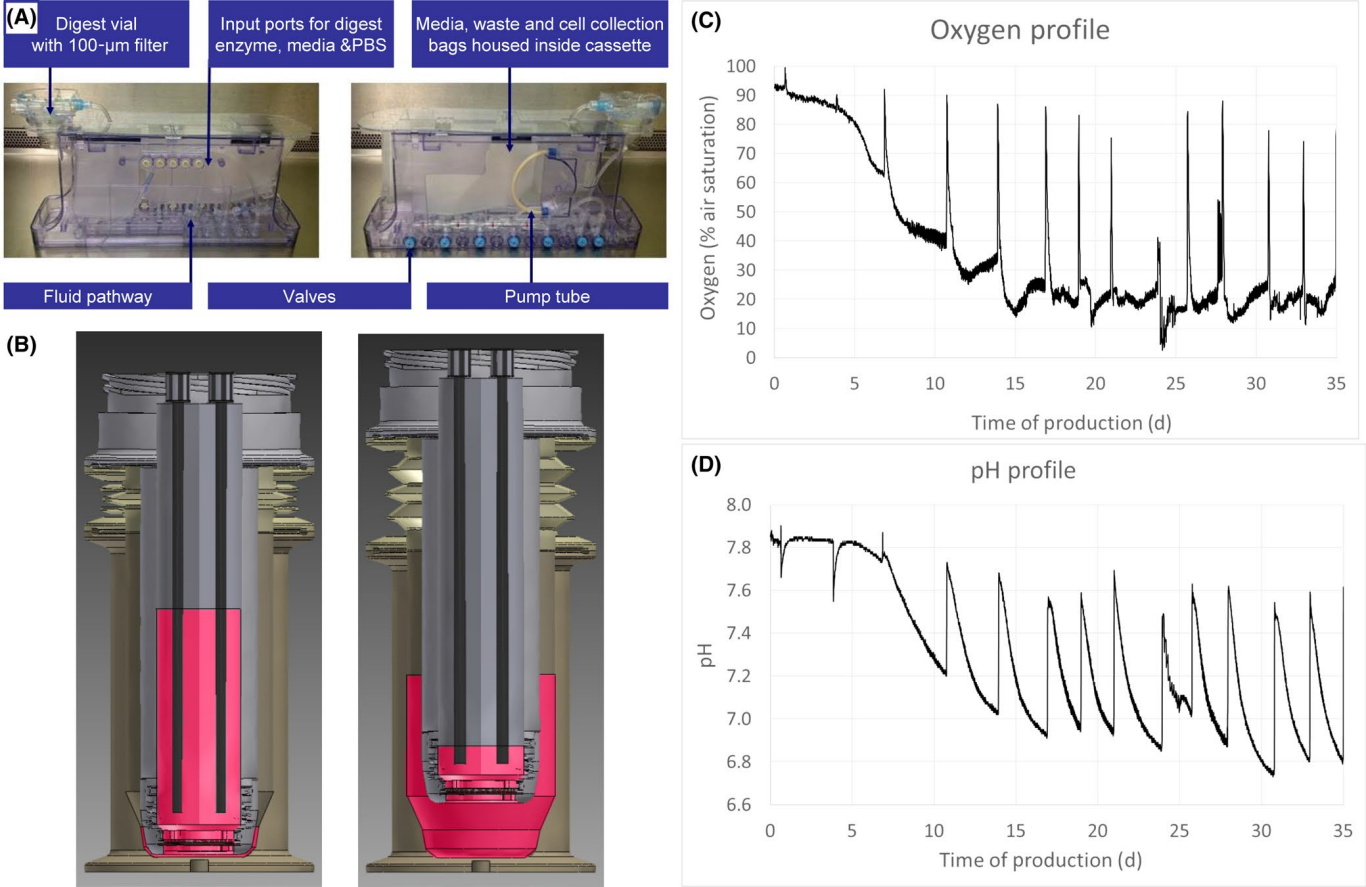
A, Cartilage digestion bioreactor. An automated tissue digest protocol was developed and converted to an automated algorithm using application‐specific Octane software. The control system operates a series of valve actuators to open and close valves on the bioreactor cassette and a peristaltic pump to deliver fluids at a range of flow rates. All biological processes are housed within a single disposable cassette. Automated protocol steps included (a) tissue washed with phosphate‐buffered saline; (b) delivery and perfusion of digestion enzyme; (c) removal of the digestion enzyme and replacement with complete medium; and (d) cell collection. B, T‐CUP perfusion bioreactor. Cell seeding, 3D expansion and differentiation were performed in a single perfusion bioreactor manufacturing module. Culture medium is forced back and forth between the inner chamber and an outer chamber of the vessel and, therefore, is perfused directly through the 3D construct. C, Oxygen and D, pH sensor data monitored throughout 5‐week culture period in the T‐CUP bioreactor. Spikes in the oxygen plot are artefacts due to the opening of the incubator door. Peaks in the pH plot are due to fluctuations in pH between the introduction of fresh medium (pH ≈ 7.6) until the next medium exchange (pH ≈ 6.8‐7.0)

The system is comprised of two bioreactor units. The *cartilage digestion bioreactor* automates and controls the cartilage tissue digestion phase Figure 2A, and the *T‐CUP perfusion bioreactor* controls the cell seeding, 3D proliferation and differentiation phases Figure 2B. The digestion bioreactor and T‐CUP perfusion bioreactor were installed within a declassified cleanroom at the GMP facility (Holostem Terapie Avanzate; declassified to permit the introduction of animal‐derived cells). Laboratory technicians were trained for autonomous use of the bioreactor systems as well as all required cartilage tissue engineering bioprocesses. In line with GMP guidelines, a proper quality management system had been organized for production in the GMP facility. Stringent records ensured full traceability of all materials used. The complete manufacturing process was detailed and documented in standard operating procedures and manufacturing protocols. Proper quality controls were established to ensure standardization of the process and product. Additional testing had to be performed on the starting materials prior entering the facility (eg mycoplasma on the biopsy at arrival), and availability of GMP‐grade materials and reagents was verified for future clinical applications.

### 2.3 Nasal cartilage biopsy

Prior to commencing the large animal studies, procedures for the handling, packaging and transportation of biopsies were first established, tested and validated. Additional procedures were established to facilitate tracking of each biopsy as well as a documentation system to streamline the delivery. Six weeks prior to graft implantation, a cartilage biopsy was harvested from the nasal septum of each sheep with an 8‐mm‐diameter biopsy punch. Perichondrium was scraped from the cartilage tissue with a scalpel, and biopsies were thoroughly washed with saline, blotted with sterile gauze and stored in complete medium at 4°C for transport. After harvesting the nasal septum cartilage in the veterinary clinic in Croatia, biopsies were transported under defined conditions (eg overall duration of transport, temperature of transport vehicle, monitored temperature within the transport container) to the GMP facility in Italy. Animals were kept at the clinic post‐operatively and then transported to a family farm until graft implantation. This 6‐week time period allowed for the production of the nasal chondrocyte grafts.

### 2.4 Cartilage tissue digestion

After passing defined acceptance criteria, biopsies were cut into small pieces (≈1‐2 mm) and transferred into the digest module of the digestion bioreactor. The digestion bioreactor (Octane Biotech) is based upon a patient‐scale cell therapy system, which allows automation of cell culture processes and protocols tailored to the application. A disposable cassette was customized for this study protocol and composed of three single‐use modular units: (a) digest module, (b) fluid delivery module and (c) collection module. The digest module houses the tissue biopsy and reagents during the process. The fluid delivery module allows automated delivery of solutions (PBS, collagenase and culture medium) through the digest module and collection module. The cell suspension is delivered automatically to the collection module at the end of the process. The digest bioreactor cassette is attached via non‐fluid contact to the bioreactor controller, which houses all the electronics, motor, valve actuators and software to control the system bioprocesses via the user interface. Biopsies were digested with 0.15% collagenase type II in complete medium (DMEM, 10% FBS, 0.1 mmol/L non‐essential amino acids, 1 mmol/L sodium pyruvate, 100 nmol/L HEPES buffer, 100 U/mL penicillin, 100 µg/mL streptomycin and 0.29 mg/mL l‐glutamine) within the automated digestion bioreactor for 20 hours at 37°C. Isolated chondrocytes were harvested from the bioreactor collection module, counted and transferred to the T‐CUP perfusion bioreactor.

### 2.5 Cell seeding, proliferation and differentiation

Nasal chondrocyte grafts were generated in the T‐CUP bioreactor using a streamlined bioreactor‐based process as previously described.^6^ Briefly, the limited number of primary chondrocytes, freshly isolated from the small cartilage biopsy, was seeded and extensively expanded directly within the 3D scaffold in the bioreactor, therefore bypassing the conventional method of 2D expansion in flasks. The T‐CUP perfusion bioreactor system (Cellec Biotek AG) is composed of three main components: (a) vessel, (b) drive unit and (c) control unit. The T‐CUP vessel houses the scaffold and culture medium. Cells are seeded and cultured within the vessel. Sensor spots to measure the oxygen and pH of the culture medium are integrated within the vessel. The T‐CUP drive unit controls the movement of the vessel which induces perfusion of culture media through the scaffold. The T‐CUP control unit controls the drive unit via the user interface. Inducing perfusion of cell suspension and/or culture medium by movement of the scaffold itself, the set‐up and all associated handling by the operator could be considerably simplified, with increased product safety and maximized opportunities for upscaling.

Chondrocytes were seeded into the bilayered biomimetic osteochondral scaffold 25 mm in diameter and 5 mm in thickness under alternating perfusion flow within the bioreactor at a perfusion rate of 1 mm/second for 16 hours in 50 mL of complete medium. Following the perfusion cell seeding phase, cell‐seeded scaffolds remained within the bioreactor, and culture medium was replaced with “proliferating medium” (complete medium supplemented with 1 ng/mL TGFβ1 and 5 ng/mL FGF‐2) to expand the cells directly within the scaffold. Constructs were perfused for 3 weeks at a perfusion rate of 100 µm/s with two medium exchanges per week. Following the 3D proliferation phase, culture medium was replaced with “differentiating medium” (complete medium supplemented with 10 ng/mL TGFβ1, 1 IU/mL insulin and 0.1 mmol/L ascorbic acid 2‐phosphate) and constructs were cultured in the bioreactor for an additional 2 weeks with medium exchanges three times per week. Throughout the proliferation and differentiation phases, pH and oxygen levels in the medium were monitored (measurements acquired every 10 minutes) with chemo‐optic sensors (PreSens GmbH) integrated into the T‐CUP bioreactor vessel. Following 5 weeks of production, half of each engineered nasal chondrocyte‐based graft was harvested for histological assessments and the other half was transported from the GMP facility to the veterinary clinic using the transportation conditions and procedures established for biopsy transport.

### 2.6 Acute defect model

The large animal study based on an acute defect model Figure S1 received approval from local research ethics committee (University of Zagreb, Faculty of Veterinary Medicine, Class 640‐01/14‐17/49, Reg. No. 251‐61‐01/139‐14‐4) and the national authorities (Ministry of Agriculture of Croatia; Class UP/I‐322‐01/14‐01/84, Reg. No. 525‐19/0255‐14‐4). Skeletally mature female German Wustenrot sheep, between the ages of 1 and 3 years old (57.2 ± 13.7 kg), were included in the studies. Sixteen animals were randomly assigned to two experimental groups: cell‐based grafts manufactured in the bioreactor (“BR,” n = 4 sheep at 3M, n = 4 sheep at 12M) and cell‐free scaffold implants (“CFS,” n = 4 sheep at 3M, n = 4 sheep at 12M).

### 2.7 Chronic defect model

The large animal study based on a chronic defect model Figure S1 received approval from local research ethics committee (University of Zagreb, Faculty of Veterinary Medicine, Class 640‐01/12‐17/99, Reg. No. 251‐61‐01/139‐12‐2) and the national authorities (Ministry of Agriculture of Croatia; Class UP/I‐322‐01/13‐01/79, Reg. No. 525‐10/0255‐13‐3). Nine animals (54.9 ± 17.3 kg) were randomly assigned to two experimental groups (BR with n = 2 sheep at 3M, n = 2 sheep at 12M; and CFS with n = 2 sheep at 3M, n = 3 sheep at 12M).

- *1st joint surgery—defect creation*: Six weeks prior to graft implantation, a cartilage biopsy was harvested from the nasal septum of each sheep as described above. In addition, cartilage defects were also created at this time on the load bearing surfaces of medial and lateral femoral condyles with a 4‐mm‐diameter biopsy punch. Special care was taken not to damage the subchondral bone.
- *2nd joint surgery—defect repair*: Defects on the medial and lateral femoral condyles, which had chronified over the 6‐week period, were converted to osteochondral defects 6 mm in diameter and 5 mm in depth using a standard instrument for mosaicplasty (COR, DePuy Synthes). Evidences of joint inflammation in terms of mild synovial oedema and hyperaemia were observed at the time of scaffold/tissue implantation in the chronic defect model. Prior to implantation, defects were washed with saline. Implantation of BR grafts and CFS implants, post‐operative care and harvesting of the explants were similar as described in the acute defect model.

### 2.8 Anaesthesia

For identification, ear tags were applied and microchips were placed under the skin at the back of the neck between the shoulder blades on the dorsal midline. Animals had food removed 24 hours before surgery and water removed 8‐12 hours ahead. Sheep were weighed and sedated with an intramuscular injection of xylazine 0.1 mg/kg (Xylapan, Vetoquinol) and ketamine 7.5 mg/kg (Narketan, Vetoquinol). General anaesthesia was induced with an injection of diazepam 0.2 mg/kg (Apaurin, Krka‐Farma doo) in the antebrachial vein, and if necessary, thiopental was also administered intravenously at a dosage of 5‐10 mg/kg. An endotracheal tube was placed in the trachea, and anaesthesia was maintained via inhalational of a mixture of 1%‐2% isoflurane (Forane, Abbott) and oxygen. Intraoperative analgesia was assured with continuous administration of fentanyl 0.2 mg/kg/min (Fentanyl injections, Janssen Pharmaceutica NV). Post‐operative analgesia was assured with meloxicam 15 mg/1.5 mL (Movalis, Boehringer Ingelheim, Croatia) in a bolus dose of 0.2 mg/kg intramuscular followed by a dose of 0.1 mg/kg intramuscular once a day. Antibiotic prophylaxis was given via intravenous cefazolin 20mg/kg (Zepilen, Medochemie/Medicuspharma).

### 2.9 Graft implantation

Each stifle was physically examined for any abnormalities while anaesthetized. The animal was placed in a dorsal recumbence position, and following surgical preparation, the right stifle joint was opened via a medial parapatellar approach. The Hoffa was incised to facilitate visualization of the joint, and the knee was flexed to make the medial femoral condyle visible. For the visualization and approach to the lateral femoral condyle, a lateral parapatellar mini‐arthrotomy was performed. After exposure, osteochondral defects measuring 6 mm in diameter and 5 mm in depth were created with a standard instrument for mosaicplasty (COR, DePuy Synthes) on both medial and lateral femoral condyles. In the BR group, medial and lateral defects were both treated with autologous nasal chondrocyte‐based grafts. In the CFS group, medial and lateral defects were both treated with cell‐free scaffolds. BR grafts and CFS implants were cut to a cylindrical shape with a 6‐mm‐diameter biopsy punch and implanted into the defects using a press‐fit method. No additional fixation was used. After the implantation, the joint was cycled through a range of motion to ensure a satisfactory rim fixation of the implanted graft or scaffold, following which the joint was closed by standard surgical procedures. Post‐operatively, animals were allowed to bear full weight, but kept in small pens for 5 days to reduce ambulation. After 5 days in the clinic, animals were transferred to a family farm with no ambulation restrictions.

### 2.10 Euthanasia and necropsy

Following 3 months or 12 months, animals were sedated according to anaesthesia protocols with intramuscular administration of ketamine and xylazine and subsequently euthanized with intravenous administration of T61. To harvest the treated defects, condyles were cut with an oscillation saw. From each condyle, an osteochondral tissue block containing the defect and surrounding cartilage was cut to a size of 15 × 10 × 10 mm and subsequently divided into two halves.

### 2.11 Explant characterization

#### 2.11.1 ICRS macroscopic scoring

Before explantation, two photographs of the exposed condyles were taken in situ. After explantation, four additional photographs were taken of each condyle. Three blinded orthopaedic surgeons, experienced in using ICRS macroscopic scoring system, independently scored the photographs.

#### 2.11.2 Histology and Immunohistochemistry

Osteochondral tissue samples for histology and immunohistochemical analysis were fixed in 4% PFA, decalcified in 15% EDTA, dehydrated in ethyl alcohol and embedded in paraffin. Deparaffinized and rehydrated 5‐µm sections were stained with haematoxylin and eosin (H&E), safranin‐O and picrosirius red. Slides were examined under a bright field and polarized light microscope, and scored according to ICRS II histology criteria^10^ by two blinded and independent observers. For each parameter, nine slides were examined per explant (three slides per histological stain) to determine the score.

For the immunohistochemical detection of collagen type I, collagen type II and aggrecan, 5‐µm sections were deparaffinized and rehydrated, and antigen retrieval performed by incubation with 0.1% proteinase K (Agilent Technologies), 0.1% proteinase K and 2.5% hyaluronidase, or 0.2 U/mL chondroitinase ABC (Sigma‐Aldrich), respectively. Sections were washed in PBS and endogenous peroxidase quenched with 3% H_2_O_2_. Sections were then blocked with 10% goat serum and incubated with anti‐collagen I (1:100 dilution; #5D8‐G9, Abcam), anti‐collagen II antibody (1:20 dilution; #II‐II6B3, Developmental Studies Hybridoma Bank), anti‐aggrecan, (1:50 dilution; #CSPG1, Acris Antibodies GmbH) or an isotype‐specific immunoglobulin at the corresponding concentration (negative control). Sections were washed in PBS and incubated with a secondary peroxidase‐conjugated antibody (Dako REAL Envision Detection System kit, Agilent Technologies) following the manufacturer's instructions. Sections were incubated with 3′,3′ diaminobenzidine tetrahydrochloride (DAB) and counterstained with haematoxylin. Normal articular cartilage and subchondral bone were used as positive controls.

#### 2.11.3 Histological quantification

To quantify immunohistochemical staining of collagen type II, slides were scanned with NanoZoomer 2.0‐RS. The reference area was set to 32.5 mm^2^, corresponding to the size of the defect. Collagen type II positive area was measured with NDPview2 software. Quantification of immunohistochemical staining for collagen type I was assessed based on a weighted score. First, the intensity of staining was defined with grades 0‐5 (0 = no positive staining, 1 = very mild positive staining, 2 = mild positive staining, 3 = moderate positive staining, 4 = strong positive staining, 5 = very strong positive staining). Slides were scanned with NanoZoomer 2.0‐RS, and the area of each grade was measured with NDPview2 software. Scores were calculated from the sum of each grade multiplied by its area. Immunohistochemical staining of aggrecan was quantified with scoring grades from 0 to 6 (0 = no positive staining, 1 = very mild positive staining, 2 = mild positive staining, 3 = moderate positive staining, 4 = strong positive staining, 5 = very strong positive staining as in normal cartilage, 6 = very strong staining that extends outside the cartilage layer into subchondral bone).

### 2.12 Statistics

Quantitative data are expressed as the mean ± standard deviation (SD). To analyse differences, independent‐samples *t* test was used and Hedges g effect size was calculated using the formula from Ellis, P.D. 11 Welch‐Satterthwaite correction was used for variables with unequal variances between groups, as determined by Levene's test. Due to the number of comparisons in ICRS II histology scores, the Benjamini‐Hochberg procedure was used to control for false discovery rates^12^ to determine statistical significance. For other tests, *P* values >0.05 were considered statistically significant. All analyses were performed with IBM SPSS Statistics (version 22.0. Armonk, IBM Corp).

## 3 RESULTS

### 3.1 Bioreactor‐based manufacture

All of the bioreactor‐based production runs, for both animal studies, were successfully completed. Digestion of nasal cartilage biopsies in the automated digestion bioreactor provided a yield of 28,400 ± 19,100 nasal chondrocytes per biopsy. Preliminary experiments showed that nasal chondrocytes were seeded predominantly within the chondral region of the bilayered scaffold (data not shown). Therefore, scaffolds were seeded at an average density of 2.9E + 04 chondrocytes/cm^3^ scaffold volume (accounting for the volume of the chondral layer only). Oxygen measurements acquired throughout the production process showed a large drop in the oxygen level over 2 to 3 weeks of the 3D proliferation phase (Figure 2C, 100% air saturation corresponding to 21% oxygen concentration). Oxygen levels then reached a plateau of 10%‐30% air saturation levels during the 2‐week differentiation phase. pH profiles were consistent with oxygen profiles, showing a large drop in pH over the 3D proliferation phase Figure 2D. Since culture medium was exchanged batchwise, large pH fluctuations can be observed between the introduction of fresh medium (pH ≈ 7.6) until the next medium exchange (pH ≈ 6.8‐7.0).

Following 5 weeks of production, engineered grafts contained extracellular matrix that loosely filled the pores of the chondral layer of the scaffold. Although some cells could be observed within the bone layer of the scaffold, cells were predominately distributed within the chondral layer Figure S2, since the chondral layer of the scaffold is structurally more favourable to cell invasion, while the more compact mineralized layer is rather cell‐occlusive.

### 3.2 Acute defect model

Grafts produced in the established bioreactor‐based manufacturing system were first tested in an acute defect model, based on freshly created osteochondral defects. Surgical wounds healed well after arthrotomy, with no signs of oedema, inflammation or wound dehiscence. Upon explantation, there were no signs of delamination or dislocation of any grafts or implants. Unfortunately, three sheep in the BR group died (two in the 3‐month group and one in the 12‐month group). Upon examinations by an independent examiner, reasons of death were found to be unrelated to the surgical procedures or to the implants.

#### 3.2.1 Macroscopic evaluation

**Figure 3:**
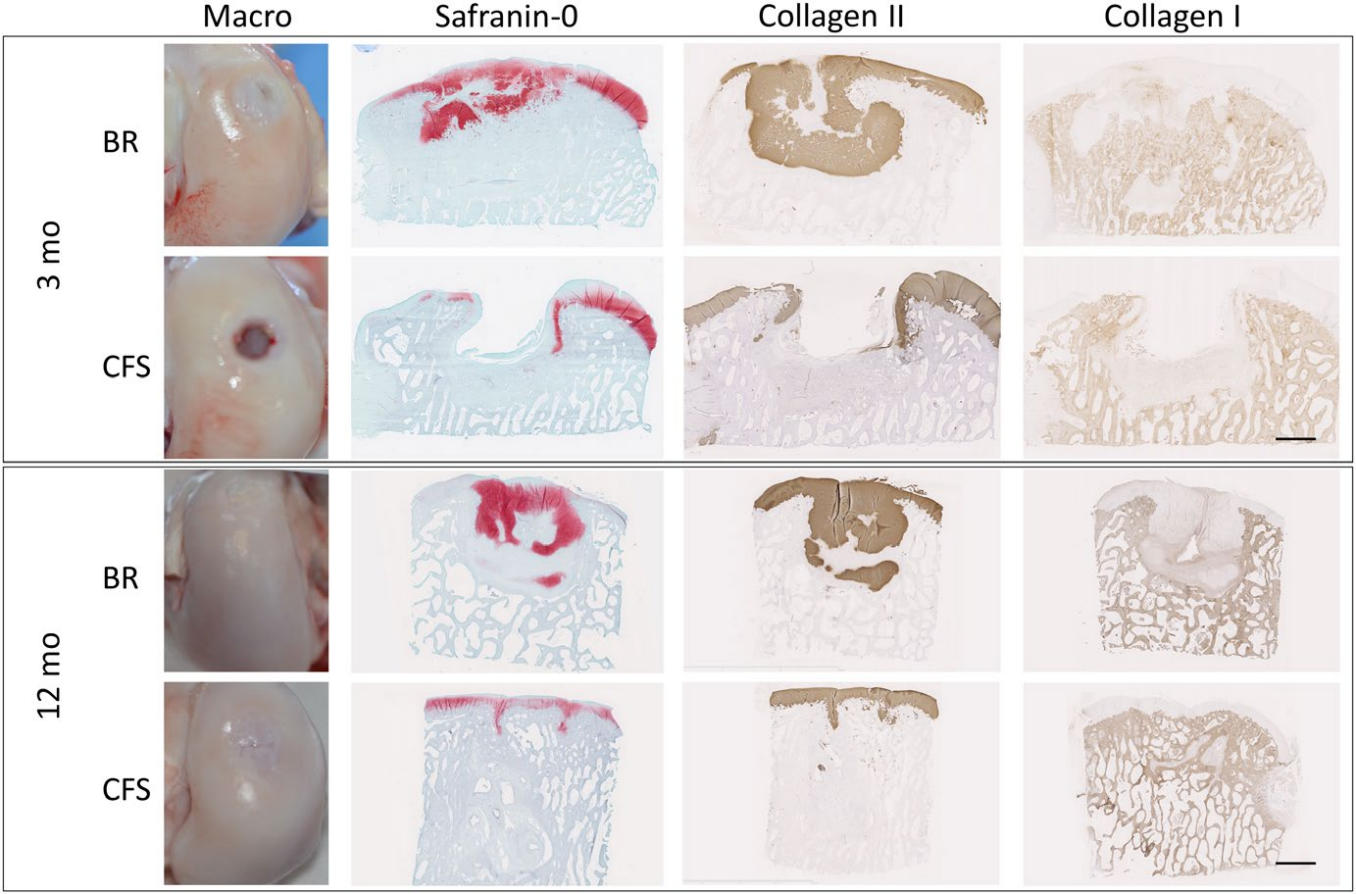
Explants from acute defects treated with bioreactor (BR) manufactured grafts and cell‐free scaffold (CFS) implants. Scale bars indicate 2 mm

At 3 months, BR explants had better gross morphology than CFS explants Figure 3 ^,^ with significantly higher ICRS overall macroscopic scores (BR: 7.3 ± 0.82, n = 4 vs CFS: 2.8 ± 1.5, n = 8; *P* = 0.0002). BR defects were filled with glossy white tissue, while CFS defects were partially filled with granulation/fibrous tissues. At 12 months, cartilage repair appeared macroscopically better for both groups compared to the 3‐month time point. Repair tissue filled the defects and appeared to be integrated with the surrounding cartilage for both groups. The surface of BR defects appeared smoother with less fissures and had significantly higher ICRS macroscopic scores (BR: 9.1 ± 0.8, n = 6; vs CFS: 6.8 ± 1.9, n = 8; *P* = 0.014) as compared to CFS defects.

#### 3.2.2 Histological evaluation

**Figure 4:**
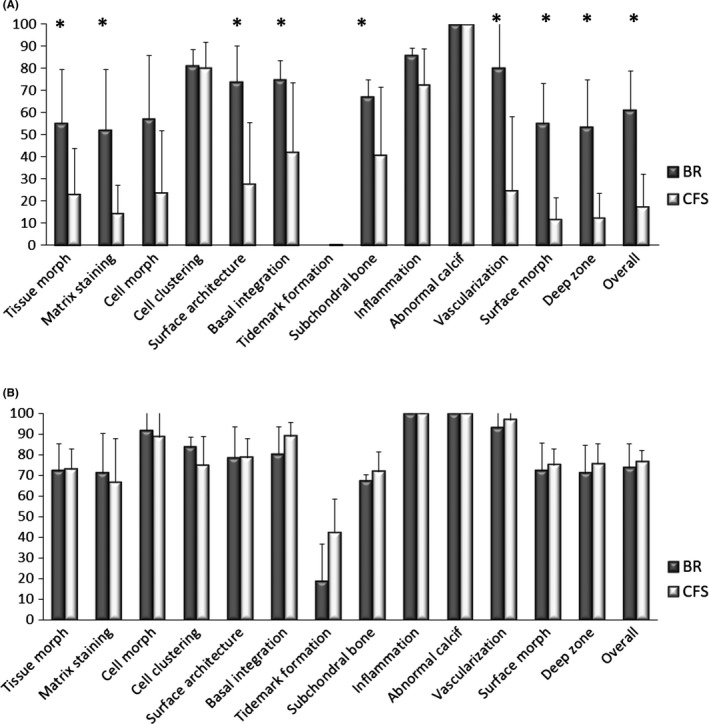
ICRS II histology scores of explants from acute defects treated with bioreactor (BR) manufactured grafts and cell‐free scaffold (CFS) implants. A, 3‐mo explants. B, 12‐mo explants. Data are presented as mean + SD. (*indicates statistically significant difference between BR and CFS)

At 3 months, ICRS II histological scores were significantly higher for BR‐treated defects than CFS for the parameters: tissue morphology (*P* = 0.037), matrix staining (*P* = 0.008), surface architecture (*P* = 0.013), basal integration (*P* = 0.023), subchondral bone (*P* = 0.049), vascularization (*P* = 0.019), surface morphology (*P* = 0.012), deep zone morphology (*P* = 0.024) and the overall score (*P* = 0.001) Figure 4A. BR‐treated defects were filled with hyaline cartilage, positively stained for safranin‐O and collagen type II Figure 3, as well as aggrecan. Chondrocytes were embedded within lacunae, although with a random organization. Cartilaginous repair tissue in BR defects appeared to be integrated with the surrounding native cartilage. CFS‐treated defects were only partially filled with granulation and fibrous tissues that stained poorly for safranin‐O, collagen type II and aggrecan. The partial filling resulted in cavities at the centre of these defects. CFS defects contained abundant blood vessels, as well as leucocytes and macrophages, consistent with wound healing and tissue remodelling. Residual scaffold material was detected in the defects of CFS explants Figure S3. In contrast to BR defects, cartilage‐cartilage integration was not observed for the CFS group. Although no tidemark could be observed at 3 months for either group, subchondral bone regeneration was more advanced in BR defects than in CFS. Repair tissue in BR defects was integrated with surrounding bone and contained areas of active ossification. In contrast, the subchondral region in CFS defects was only partially filled with loose granulation tissue, with few areas of active ossification.

The quantified area of collagen type II staining was significantly larger for BR vs CFS in the chondral region of the repair tissues (3.5 ± 1.2 vs 1.6 ± 0.71 mm^2^; *P* = 0.006). Although histological scoring of aggrecan staining was higher for BR vs CFS in the chondral region (3.0 ± 2.21 vs 0.9 ± 1.68; *P* = 0.098), the difference was not statistically significant. The scoring of collagen type I staining was similar for BR and CFS in the chondral region (0.25 ± 0.21 and 0.44 ± 0.35) as well as in the subchondral region (1.75 ± 0.76 and 2.49 ± 1.27).

At 12 months, there were no significant differences in the ICRS II histological scores between the BR and CFS explants Figure 4B. However, nearly all scores were higher at 12 months compared to the 3‐month time point. BR and CFS defects were filled with hyaline matrix, positively stained for safranin‐O, collagen type II and aggrecan, and a columnar organization of chondrocytes. While the safranin‐O‐stained area appears larger *throughout* 3‐month BR explants than *throughout* 12‐month BR explants Figure 3, the chondral region was assessed separately from the subchondral region for histological quantifications as well as for specific parameters of the ICRS II histological scores. Virtually, no collagen type I staining was observed in the chondral layer of either BR or CFS groups. The repair tissue in both groups had a smooth surface and appeared integrated with the surrounding cartilage tissue, with no discernible borders. Hyaline cartilage extended into the subchondral region of the BR group, whereas collagen type I with a bone‐like structure filled the subchondral region of the CFS group. Large areas of cystic fibrous tissue were present in the subchondral bone of the CFS group Figure S4. The presence of pseudocysts was observed in the subchondral region of both BR and CFS defects.

The quantified area of collagen type II staining (3.8 ± 1.1 vs 3.8 ± 0.97 mm^2^) and the histological scoring of aggrecan staining (2.3 ± 1.2 and 2.7 ± 1.5) were similar for BR vs CFS in the chondral region of the repair tissues at 12 months. Negligible collagen type I staining was observed in the chondral region of either group. Collagen I scoring of the subchondral region was significantly lower for BR vs CFS (2.5 ± 0.93 vs 4.1 ± 0.86, *P* = 0.005).

### 3.3 Chronic defects

Following the promising results obtained with bioreactor‐produced engineered grafts in the acute defect model, we next aimed to assess the grafts in a more challenging and more clinically relevant model of a chronic defect.

#### 3.3.1 Macroscopic evaluation

**Figure 5:**
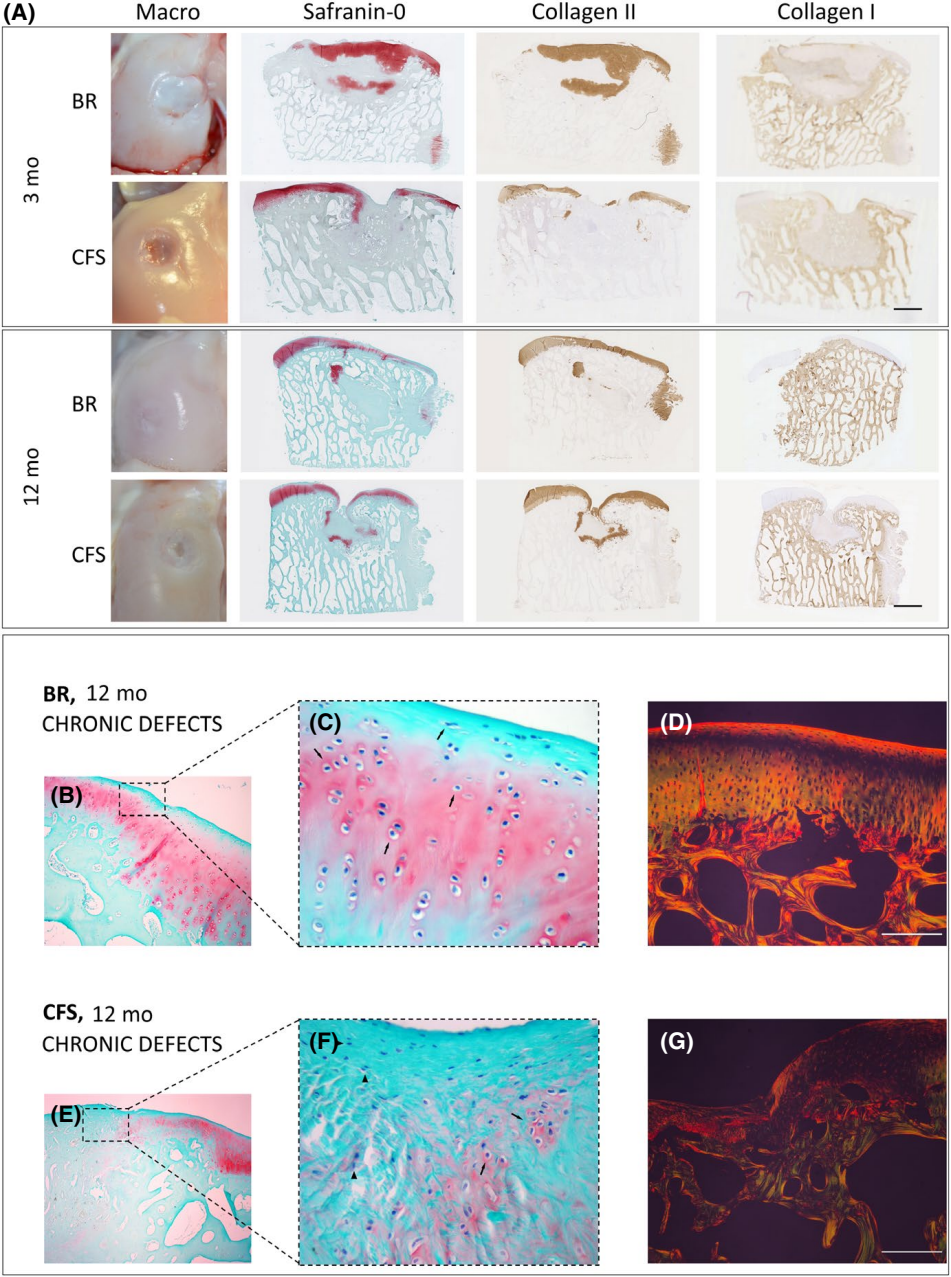
Explants from chronic defects treated with bioreactor (BR) manufactured grafts and cell‐free scaffold (CFS) implants. A, Macroscopic and histological assessments after 3 and 12 mo. Scale bar indicates 2 mm. B‐G, 12‐mo explants from chronic defects. B, C, In BR chronic defects, we observed restoration of articular cartilage with chondrocytes (arrows) in zonal organization typical for articular cartilage. E, F, CFS defects healed with fibrous tissue mixed with hyaline cartilage. Both chondrocytes (arrows) and connective tissue cells (arrowheads) are present, but with no tissue organization. D and G Picrosirius‐stained images observed under polarized light microscope show excellent cartilage‐to‐cartilage integration in BR explants, with homogenous fibril organization across the defect. Poor cartilage healing is observed in CFS explants, with thick non‐organized fibres. B, C, E, F, Safranin‐O staining. D, G, Picrosirius red staining as observed under polarized light microscopy. Scale bar indicates 100 µm

At 3 months, BR and CFS defects were partially filled with hyaline and fibrous tissues Figure 5A^,^ with similar ICRS macroscopic scores (BR: 4.2 ± 2.0, n = 4 and CFS: 4.8 ± 1.2, n = 4). One of the BR defects was nearly completely filled. Tissue formed in the defects of both BR and CFS appeared to be integrated with the surrounding cartilage but areas with demarcation borders were noticeable. At 12 months, cartilage repair appeared better for both groups compared to the 3‐month time point. Unfortunately, due to reasons unrelated to the surgery or to the implants, only one sheep in the BR group survived the full 12 months, and therefore, no statistical analyses were performed for the 12‐month time point. Nevertheless, for the two BR defects assessed, ICRS macroscopic scores were higher than for any of the six CFS defects (BR: 10.7 ± 0.6, n = 2; vs CFS: 5.5 ± 2.01, n = 6). BR defects were completely filled with glossy white hyaline‐like tissue, with a smooth surface and which was integrated with the surrounding cartilage tissue. In contrast, CFS explants contained fibrous tissue only partially filling the defect, with visible fissures and uneven edges.

#### 3.3.2 Histological evaluation

**Figure 6:**
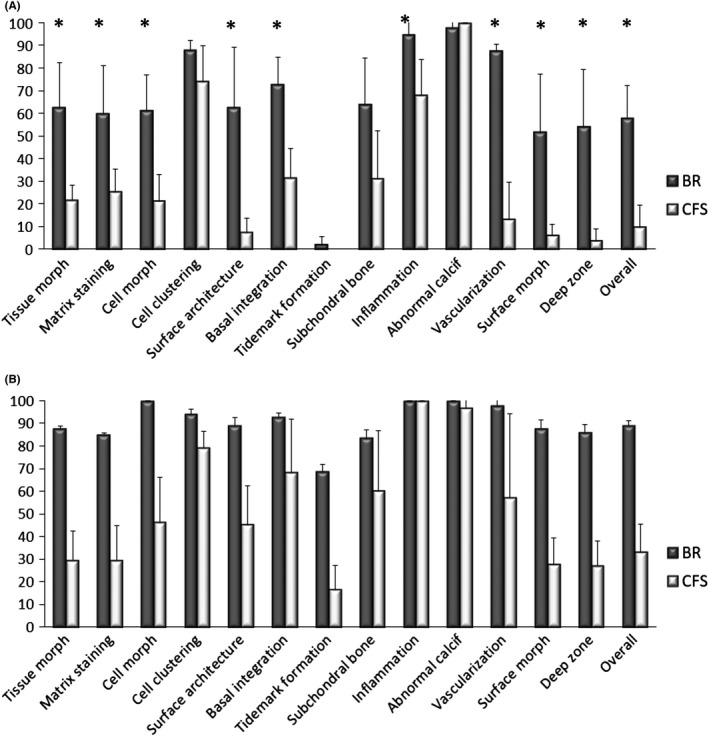
ICRS II histology scores of explants from chronic defects treated with bioreactor (BR) manufactured grafts and cell‐free scaffold (CFS) implants. A, 3‐mo explants. B, 12‐mo explants. Data are presented as mean + SD. (*indicates statistically significant difference between BR and CFS)

At 3 months, ICRS II histological scores were significantly higher for BR‐treated defects than for CFS defects for the parameters: tissue morphology (*P* = 0.0079), matrix staining (0.0247), cell morphology (*P* = 0.0066), surface architecture (*P* = 0.0067), basal integration (*P* = 0.0035), inflammation (*P* = 0.0357), vascularization (0.0023), surface morphology (*P* = 0.129), deep zone assessment (*P* = 0.0078) and overall score (*P* = 0.0015) Figure 6A. BR‐treated defects were filled with hyaline cartilage, positively stained for safranin‐O and collagen type II Figure 5A, as well as aggrecan. Cartilaginous repair tissue in BR defects appeared to be integrated with the surrounding native cartilage. CFS defects were infiltrated with abundant blood vessels and were only partially filled with granulation and fibrous tissues that stained poorly for safranin‐O, collagen type II and aggrecan. The partial filling resulted in cavities at the centre of the defects, with safranin‐O staining of CFS explants predominately localized near the border region of the repair tissue and surrounding native cartilage. Cartilage‐cartilage integration was not observed in the CFS explants, having a clear border and demarcated transition between the repair tissue and surrounding native cartilage. As in the acute defect model, residual scaffold material was observed in the CFS group Figure S3. Repair tissue in the subchondral bone region of BR explants had visible areas of ossification and appeared to be integrated to surrounding native bone. In CFS explants, repair tissue in the subchondral bone region was predominately hypervascular and loose granulation tissue with only few areas of ossification. Macrophages and leucocytes were present in both groups but much more prevalent in CFS explants.

The quantified area of collagen type II staining (3.3 ± 1.6 vs 3.2 ± 1.8 mm^2^) and the histological scoring of aggrecan staining (1.2 ± 0.8 vs 1.0 ± 1.7) were similar for BR and CFS in the chondral region of the repair tissues. Weighted scoring of collagen type I staining was similar for BR and CFS in both the chondral (0.1 ± 1.3 vs 0.4 ± 0.3) and subchondral (1.8 ± 1.0 vs 3.0 ± 1.0) regions.

At 12 months, defect repair in both the BR and CFS groups had improved compared to the 3‐month time point. For the two BR defects assessed, ICRS II histological scores were higher than for the six CFS defects for the parameters: tissue morphology, matrix staining, cell morphology, chondrocyte clustering, surface architecture, tidemark formation, surface, deep zone and overall score Figure 6B. The chondral layer of BR defects was comprised of a smooth layer of hyaline‐like cartilage, positively stained for safranin‐O, collagen type II and aggrecan. Interestingly, chondrocytes were embedded within lacunae organized in a columnar distribution resembling native cartilage Figure 5B, 5. The articular surface was completely covered with a continuous hyaline cartilage layer, with no discernible border between the repair tissue and the surrounding native cartilage Figure 5D. Repair tissue within the chondral region of CFS defects was comprised primarily of fibrous tissue Figure 5E, 5. Fibrocartilage was present only near the defect edges, which only faintly stained for safranin‐O, collagen type II and aggrecan. In addition, blood vessels were observed in the chondral region of all CFS defects. Upward migration of subchondral plate was observed in BR explants as well as the formation of the tidemark between the cartilage and subchondral bone regions. In contrast, subchondral bone in CFS defects regenerated circumferentially with fibrous tissue present in the central subchondral region. Moreover, cysts were present in the subchondral region of most CFS explants.

The quantified area of collagen type II staining (3.4 vs 2.3 mm^2^) and scoring of aggrecan staining (4.5 ± 0.4 vs 0.4 ± 0.5) were larger for BR vs CFS in the chondral region at 12 months. Weighted scoring of collagen type I was similar for BR and CFS in both the chondral (0.0 ± 0.0 vs 0.3 ± 0.6) and subchondral (4.7 ± 0.5 vs 4.0 ± 0.9) regions.

## 4 DISCUSSION

In this work, we have established a bioreactor‐based manufacturing system for the production of chondrocyte‐based engineered grafts. All bioreactor technologies and cartilage tissue engineering bioprocesses were transferred to an independent GMP facility where all engineered grafts were successfully manufactured for two large animal studies. The results of these studies demonstrate the safety and feasibility of the bioreactor‐based manufacturing approach. Moreover, grafts produced in the bioreactor manufacturing system were first shown to accelerate the repair of acute osteochondral defects compared to cell‐free scaffold implants. We then demonstrated that grafts produced in the system also resulted in a faster repair in a more clinically relevant chronic defect model. The data also suggest that the bioreactor‐manufactured grafts may result in a more robust repair at a longer term point.

We have previously established a number of bioreactor technologies^3^, ^4^, ^5^ that have served as the foundation to establish a manufacturing process for the production of engineered tissue grafts in this study. Using this manufacturing platform, we have implemented an innovative streamlined process to expand the limited number of primary chondrocytes, which can be isolated from a small cartilage biopsy, directly within a porous 3D scaffold, bypassing the conventional manual process of cell expansion in tissue culture flasks.^6^ Using this process, cell seeding, 3D expansion and differentiation were all performed within a single perfusion bioreactor manufacturing module. In addition, digestion of the cartilage biopsy and preparation of the isolated cell suspension were performed in an innovative automated cartilage digestion bioreactor module. Therefore, all bioprocesses, from the introduction of the cartilage biopsy through the generation of the engineered graft, were performed in our bioreactor‐based manufacturing system. By minimizing labour‐intensive operator‐dependent procedures and controlling the manufacturing processes, our bioreactor system has the potential to (a) improve standardization of the manufacturing process and reproducibility of product quality, (b) increase safety of the process and product, (c) facilitate significant scaling of production volumes, (d) increase the cost‐effectiveness of the engineered product and (e) simplify transferability of the production to other manufacturing centres.^13^ In addition, non‐invasive sensors were integrated into the bioreactor system to monitor oxygen and pH levels throughout the manufacturing process. Sensing and online monitoring offer great advantages to provide non‐invasive and quantitative data relevant to the process and the engineered graft, providing meaningful in‐process quality controls and data on graft quality. Automated logging of our sensor data along with other key process parameters will increase traceability of the process and will therefore facilitate compliance to strict regulatory guidelines. The integrated sensors could be used in the future not only to monitor, but also to maintain pH and oxygen at predefined levels in a feedback controlled loop. This may not only improve and standardize graft quality, but would considerably improve process robustness and standardization for reduced process and product variability.

One potential limitation of our manufacturing strategy was the approach of utilizing a centralized manufacturing facility for the production of the engineered grafts. This approach imposes significant logistical and regulatory challenges, as well as high costs, simply for the transportation of biopsies and grafts between the central facility and the clinical site. On the other hand, a centralized manufacturing facility allows for highly trained operators to closely oversee the manufacturing process, to conduct in‐process controls and to release the final engineered product in accordance with well‐defined quality control release criteria. As specific assays will be required to characterize the final engineered product, it may be challenging and cost prohibitive for a de‐centralized facility to have available a qualified laboratory with qualified instruments, validated procedures and the trained operators that would be required to perform the necessary assays. In the future, it may be possible to establish a simple to use plug‐and‐play bioreactor system which could be installed into qualified clinical centres for the de‐centralized production of engineered tissues, mitigating complex logistical issues as well as automating aspects of quality control. Nevertheless, this will still require substantial expertise and investment in infrastructure at the production sites.

Engineered grafts produced in the manufacturing system were first assessed in a well‐established large animal model of cartilage repair, based on freshly created osteochondral defects.^14^ At the early observational time point of 3 months, grafts generated in the bioreactor system resulted in a better repair than cell‐free implants as assessed by ICRS macroscopic criteria and by 9 out of the 14 parameters of the ICRS II histological scoring criteria. Histological images of the best, the intermediate and the worst repair for each treatment group in the acute model are shown in Figure S5. BR‐treated defects were filled with glossy white hyaline cartilage tissue that appeared integrated with the surrounding native cartilage tissue along with more advanced subchondral bone regeneration. While we observed hyaline cartilage tissue also within the subchondral bone layer of BR defects at both 3 and 12 months, it is likely that this cartilage tissue would ultimately remodel into bone if assessed at later time points. Designed for human applications, the thickness of the implanted graft cartilage exceeded the thickness of articular cartilage in sheep stifle joint, which varies between 0.4 and 0.5 mm.^15^ More precise scaffold design to better fit cartilage thickness of the experimental animal may favour development of stable cartilage in the chondral layer only. Although differences were not detected histologically between the groups later at the 12‐month time point, clinical observations by the orthopaedic surgeons (ICRS macroscopic scores) would suggest more advanced defect healing in the BR‐treated defects. Taken together, the extent of repair observed at both time points in the acute defect model indicates great promise for the bioreactor‐generated grafts. In particular, more advanced repair at an early time point could have great implications on the clinical and commercial success of an engineered tissue graft. An accelerated repair may allow earlier post‐operative joint loading and shorter rehabilitation times, with a more rapid return of the patient to normal life activities. This would have a significant benefit in terms of both quality of life and reduction of healthcare costs, with the potential to influence claim reimbursement by health insurances and/or social systems.

Based on the promising results from the acute defect model, we next aimed to assess the bioreactor‐generated grafts in a more challenging model of a chronic defect. While the acute defect model (treatment of freshly created defects) has been long established for studying cartilage repair strategies, a patient would rarely be treated in the clinic immediately following an injury to their cartilage tissue. Therefore, to better mimic a clinical scenario, we have implemented a chronic defect model, in which defects were first created on the weight‐bearing surfaces of the sheep condyle and allowed to chronify over a 6‐week period prior to treatment with grafts and implants.^16^

Despite the highly challenging model, all chronic defects were partially filled with repair tissue at 3 months. While both treatment groups had similar gross appearances, chronic defects treated with bioreactor‐generated grafts resulted in a better repair based on 10 out of 14 parameters of the ICRS II histological scoring criteria. Histological images of the best, the intermediate and the worst repair for each treatment group in the chronic model are shown in Figure S6. BR‐treated defects were filled with hyaline cartilage that appeared integrated with the surrounding native cartilage and an ossifying subchondral region integrated with the surrounding native bone. While CFS defects remained only partially filled with fibrous tissue following 12 months, bioreactor‐treated defects were completed filled with a smooth layer of glossy white hyaline‐like tissue. Interestingly, while chondrocytes were found randomly distributed in the chondral repair tissue at the early time point, at 12 months, cells were embedded within lacunae and organized in structures resembling that of native articular cartilage. While cartilage extended into the subchondral bone with no evident tidemark at 3‐month time point, at 12 months, cartilage was present in the chondral region only—separated from the subchondral region by a discernible tidemark. Moreover, while cartilage‐cartilage integration remains a key challenge to address in cartilage repair strategies, at 12 months, in both the acute and chronic models, the repair tissues in bioreactor‐treated defects appeared well integrated with the surrounding native tissues, with no detectable borders. Finally, subchondral bone remodelling appeared nearly complete, including the formation of a discernible tidemark between the chondral and subchondral regions. As opposed to the acute defect model, there was no persistence of cartilage tissue within the subchondral bone layer of the BR defect. As reported in the literature, it is possible that the inflammatory environment in the chronic defect has facilitated the conversion of cartilage into bone.^17^

The results of the chronic model are quite impressive considering the mechanical loading and inflammatory conditions that the grafts were subjected to. Since sheep were not immobilized after surgery, the animals could walk freely post‐operatively. Our data show that BR grafts, which were implanted into high load bearing regions of the condyle and therefore subjected to mechanical loading soon after implantation, resulted in hyaline repair tissue resembling native cartilage tissue. This supports a previous in vitro study showing that nasal chondrocytes can respond positively to physical forces resembling joint loading, increasing the production of cartilaginous extracellular matrix proteins.^18^ Our results are also consistent with another in vitro study showing that nasal chondrocytes have a high capacity to recover from inflammatory conditions, suggesting that nasal chondrocyte‐based grafts could have more favourable chances to successfully engraft into the joint and regenerate the articular cartilage surface.^19^

While the level of maturation of engineered cartilage grafts, perhaps enhanced by bioreactor mechanical preconditioning, could potentially affect in vivo integration and remodelling, this hypothesis was outside the scope of this work. However, we are currently addressing this question in an ongoing multicentre phase II clinical study by comparing the clinical efficacy of mature vs immature tissue engineered grafts for the treatment of traumatic cartilage lesions in the knee (Nose to Knee II, Swissmedic 2016‐TpP‐2004). Nevertheless, in this current manuscript we describe that the bioreactor‐generated grafts could be safely implanted into high load bearing sites, subjected to mechanical loading soon after implantation, and result in hyaline repair tissue resembling native cartilage. If engineered cartilage grafts can be generated with sufficient properties to meet defined quality criteria without the use of mechanical preconditioning, bioreactor automation requirements can be greatly simplified, thereby facilitating the development of a more compact, user‐friendly and cost‐effective bioreactor‐based manufacturing system—facilitating clinical translation.

## 5 CONCLUSION

Conventional manufacturing strategies present significant hurdles for the cost‐effective translation of cell‐based engineered grafts to the clinic. By demonstrating safety of the generated grafts in two large animal models, this work represents a pivotal step towards a regulatory compliant, bioreactor‐based clinical manufacturing strategy for human engineered cartilage implants. Moreover, in view of the significant challenges typically associated with treating advanced cartilage defects, the promising efficacy results of our approach in a chronic defect model highlight its potential applicability not only for the treatment of small focal cartilage lesions, but also for a broader range of clinical indications. Ongoing efforts are currently aimed at qualifying the bioreactor technology and validating the associated bioprocesses in preparation for a first‐in‐human clinical study.

## CONFLICT OF INTEREST

The authors declare no conflicts of interest.

## References

[1] Mumme M , Barbero A , Miot S , et al. Nasal chondrocyte‐based engineered autologous cartilage tissue for repair of articular cartilage defects: an observational first‐in‐human trial. Lancet. 2016;388:1985‐1994. 27789021 10.1016/S0140-6736(16)31658-0

[2] Trainor N , Pietak A , Smith T . Rethinking clinical delivery of adult stem cell therapies. Nat Biotechnol. 2014;32:729‐735. 25093878 10.1038/nbt.2970

[3] Wendt D , Marsano A , Jakob M , Heberer M , Martin I . Oscillating perfusion of cell suspensions through three‐dimensional scaffolds enhances cell seeding efficiency and uniformity. Biotechnol Bioeng. 2003;84:205‐214. 12966577 10.1002/bit.10759

[4] Wendt D , Stroebel S , Jakob M , John GT , Martin I . Uniform tissues engineered by seeding and culturing cells in 3D scaffolds under perfusion at defined oxygen tensions. Biorheology. 2006;43:481‐488. 16912419

[5] Santoro R , Olivares AL , Brans G , et al. Bioreactor based engineering of large‐scale human cartilage grafts for joint resurfacing. Biomaterials. 2010;31:8946‐8952. 20800280 10.1016/j.biomaterials.2010.08.009

[6] Tonnarelli B , Santoro R , Adelaide AM , Wendt D . Streamlined bioreactor‐based production of human cartilage tissues. Eur Cell Mater. 2016;31:382‐394. 27232665 10.22203/ecm.v031a24

[7] Manferdini C , Cavallo C , Grigolo B , et al. Specific inductive potential of a novel nanocomposite biomimetic biomaterial for osteochondral tissue regeneration. J Tissue Eng Regen Med. 2016;10:374‐391. 23495253 10.1002/term.1723

[8] Tampieri A , Sandri M , Landi E , et al. Design of graded biomimetic osteochondral composite scaffolds. Biomaterials. 2008;29:3539‐3546. 18538387 10.1016/j.biomaterials.2008.05.008

[9] Calabrese G , Giuffrida R , Fabbi C , et al. Collagen‐hydroxyapatite scaffolds induce human adipose derived stem cells osteogenic differentiation in vitro. PLoS ONE. 2016;11:e0151181. 26982592 10.1371/journal.pone.0151181 PMC4794180

[10] Mainil‐Varlet P , Van DB , Nesic D , Knutsen G , Kandel R , Roberts S . A new histology scoring system for the assessment of the quality of human cartilage repair: ICRS II. Am J Sports Med. 2010;38:880‐890. 20203290 10.1177/0363546509359068

[11] Ellis PD . The Essential Guide to Effect Sizes: An Introduction to Statistical Power Meta-Analysis and the Interpretation of Research Results Cambridge University Press 2010;

[12] Benjamini Y , Hochberg Y . Controlling the false discovery rate ‐ a practical and powerful approach to multiple testing. Journal of the Royal Statistical Society Series B‐Methodological. 2018;57:289‐300.

[13] Asnaghi MA , Smith T , Martin I , Wendt D . Bioreactors: enabling technologies for research and manufacturing In: van Blitterswijk C, de Boer J, eds. Tissue Engineering. Academic Press/Elsevier: Amsterdam; 2014:393‐426.

[14] Hurtig MB , Novak K , McPHERSON R , et al. Osteochondral dowel transplantation for repair of focal defects in the knee: an outcome study using an ovine model. Vet Surg. 1998;27:5‐16. 9449173 10.1111/j.1532-950x.1998.tb00092.x

[15] Frisbie DD , Cross MW , McIlwraith CW . A comparative study of articular cartilage thickness in the stifle of animal species used in human pre‐clinical studies compared to articular cartilage thickness in the human knee. Vet Comp Orthop Traumatol. 2006;19(3):142‐146. 16971996

[16] Hepp P , Osterhoff G , Niederhagen M , et al. Perilesional changes of focal osteochondral defects in an ovine model and their relevance to human osteochondral injuries. J Bone Joint Surg Br. 2009;91:1110‐1119. 19651847 10.1302/0301-620X.91B8.22057

[17] Singh P , Marcu KB , Goldring MB , Otero M . Phenotypic instability of chondrocytes in osteoarthritis: on a path to hypertrophy. Ann N Y Acad Sci. 2018;1442(1):17‐34. 30008181 10.1111/nyas.13930

[18] Candrian C , Vonwil D , Barbero A , et al. Engineered cartilage generated by nasal chondrocytes is responsive to physical forces resembling joint loading. Arthritis Rheum. 2008;58:197‐208. 18163475 10.1002/art.23155

[19] Scotti C , Osmokrovic A , Wolf F , et al. Response of human engineered cartilage based on articular or nasal chondrocytes to interleukin‐1beta and low oxygen. Tissue Eng Part A. 2012;18:362‐372. 21902467 10.1089/ten.tea.2011.0234 PMC3267974

