# Supplementary material for: Bioreactor‐manufactured cartilage grafts repair acute and chronic osteochondral defects in large animal studies
Source: Cell Prolif. 2019 Sep 6;52(6):e12653. doi: 10.1111/cpr.12653 (PMC6869519; doi:10.1111/cpr.12653)
Supplement: Supplementary file 1 [file CPR-52-e12653-s001.docx]

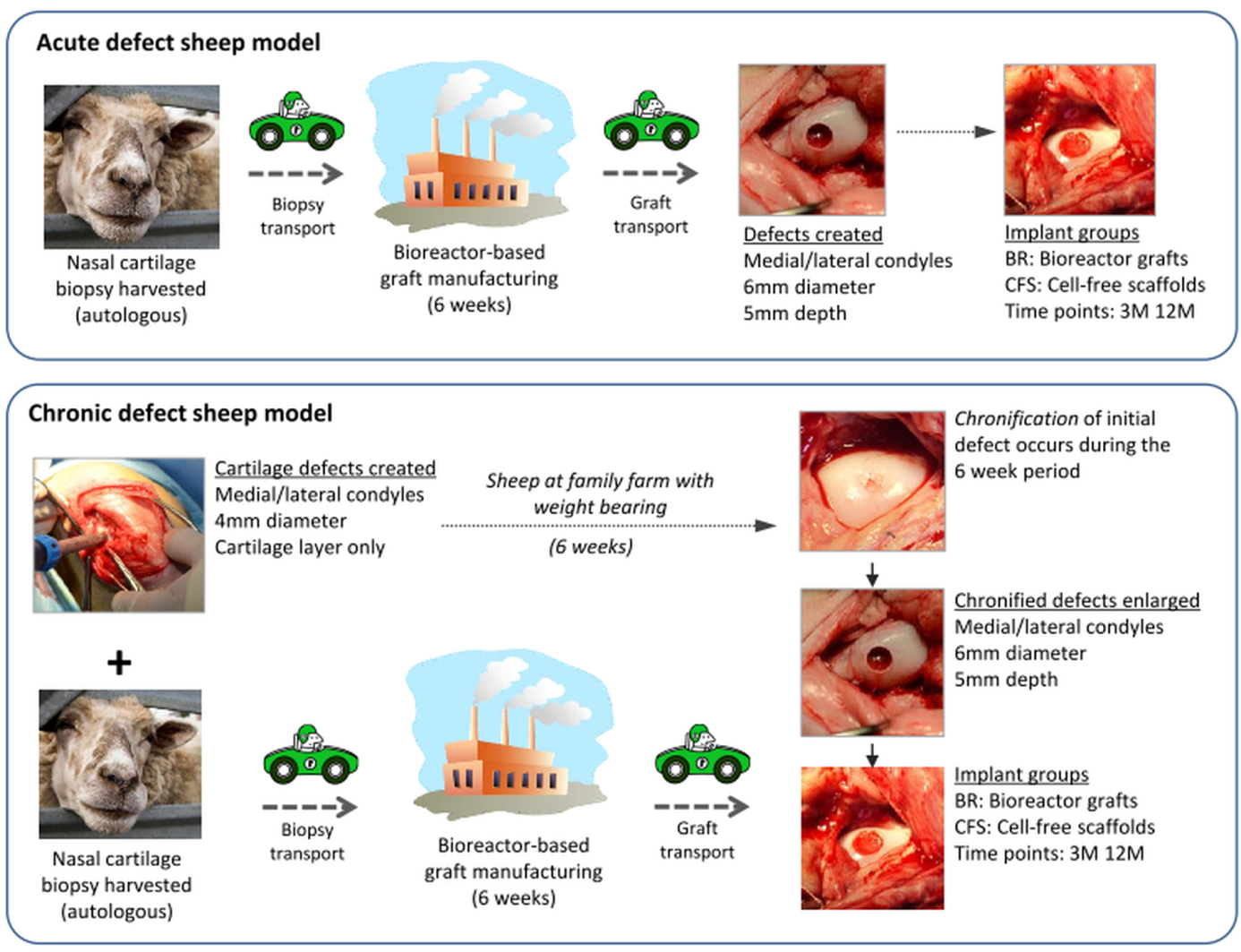


**Figure S1**. Schematic outline of the acute and chronic defect sheep models. In the acute model, osteochondral defects were created and immediately treated with bioreactor-generated grafts or cell-free scaffold implants. For the more challenging chronic model, cartilage defects were created on the loadbearing surfaces of medial and lateral femoral condyles at the same time that nasal cartilage biopsies were harvested. Following six weeks of weight bearing, these defects, which had chronified, were subsequently enlarged and treated with bioreactor-generated grafts or cell-free scaffold implants.
