# Supplementary material for: Bioreactor‐manufactured cartilage grafts repair acute and chronic osteochondral defects in large animal studies
Source: Cell Prolif. 2019 Sep 6;52(6):e12653. doi: 10.1111/cpr.12653 (PMC6869519; doi:10.1111/cpr.12653)
Supplement: Supplementary file 2 [file CPR-52-e12653-s002.docx]

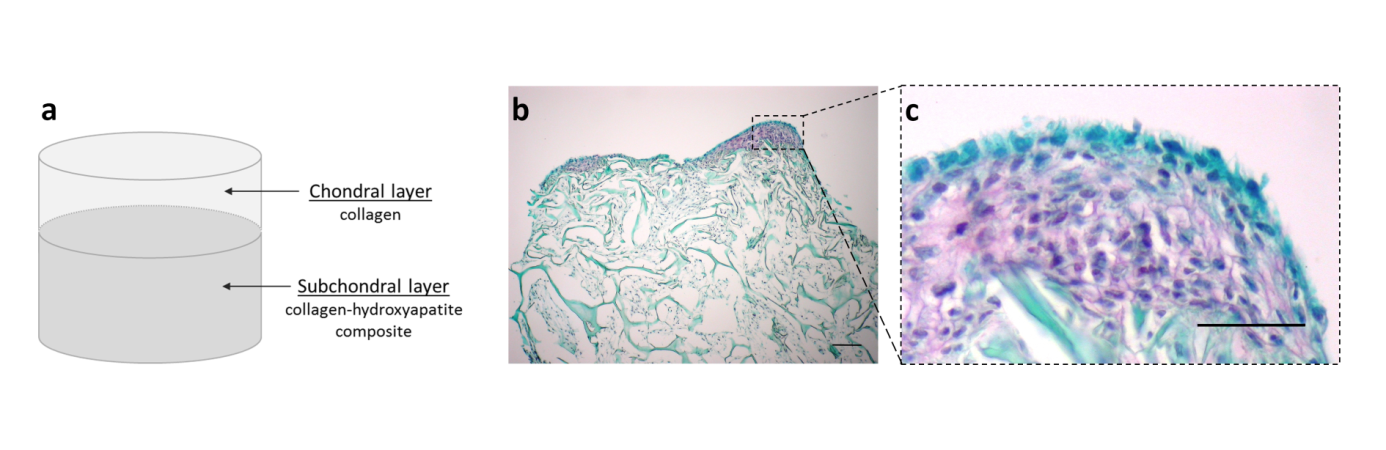


**Figure S2.** (a) Bi-layered osteochondral scaffold. (b) H&E staining of cell-based graft produced in the bioreactor-based manufacturing system.
