# Supplementary material for: Bioreactor‐manufactured cartilage grafts repair acute and chronic osteochondral defects in large animal studies
Source: Cell Prolif. 2019 Sep 6;52(6):e12653. doi: 10.1111/cpr.12653 (PMC6869519; doi:10.1111/cpr.12653)
Supplement: Supplementary file 3 [file CPR-52-e12653-s003.docx]

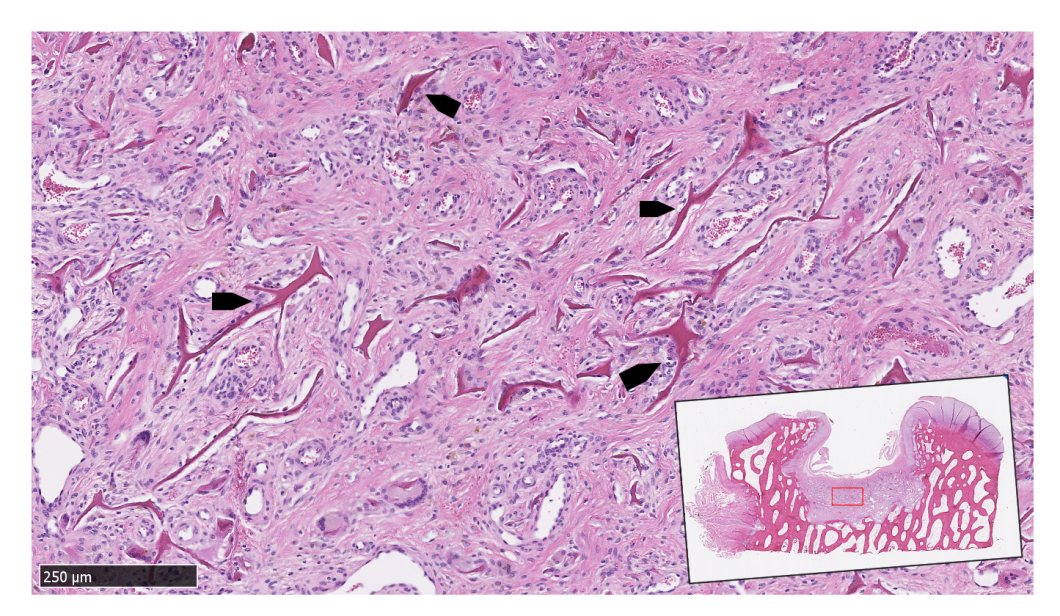


**Figure S3**. CFS explant from acute defect study shows residual scaffold material (black arrowheads). H&E staining. Scale bar indicates 250µm.
