# Supplementary material for: Bioreactor‐manufactured cartilage grafts repair acute and chronic osteochondral defects in large animal studies
Source: Cell Prolif. 2019 Sep 6;52(6):e12653. doi: 10.1111/cpr.12653 (PMC6869519; doi:10.1111/cpr.12653)
Supplement: Supplementary file 4 [file CPR-52-e12653-s004.docx]

**
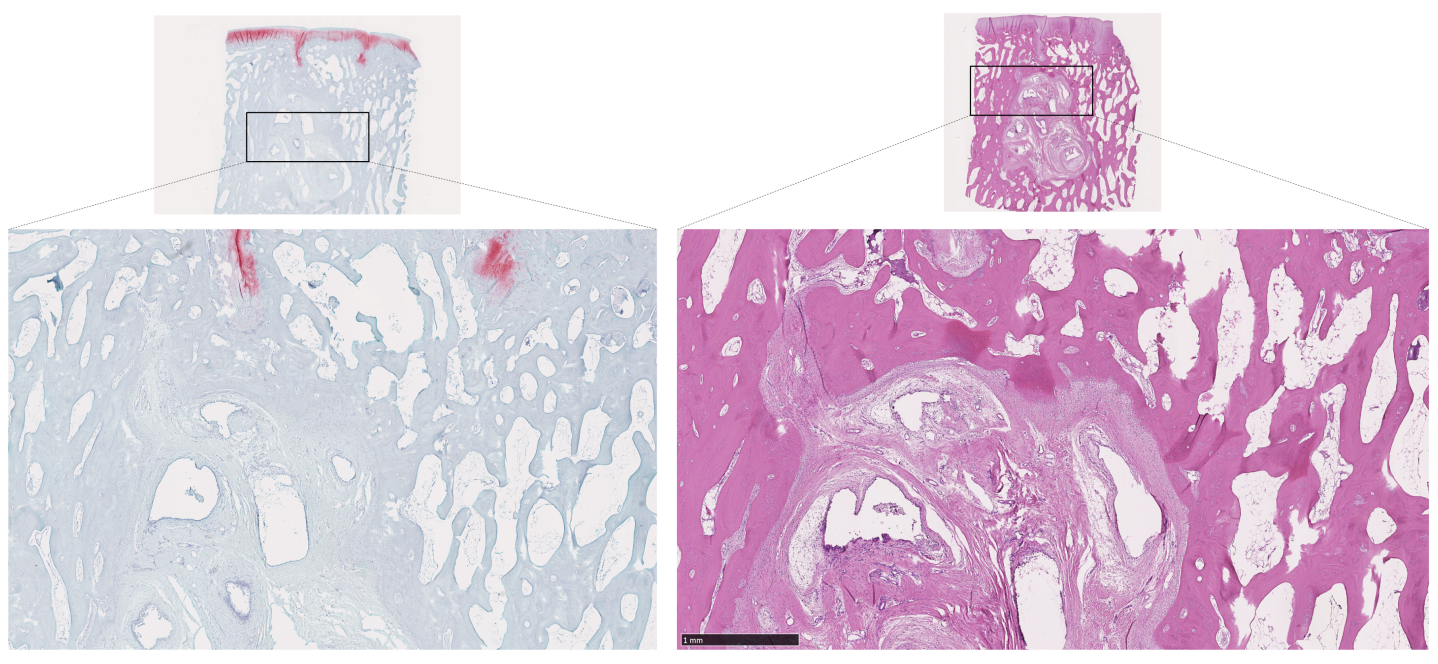
**

**Figure S4.** 12 month explants from acute defects treated with cell-free scaffold (CFS) implants. Large areas of cystic fibrous tissue are present in subchondral bone of acute CFS defects. Safranin-O staining on the left, H&E staining on the right. Scale bar indicates 1 mm.
