# Supplementary material for: Bioreactor‐manufactured cartilage grafts repair acute and chronic osteochondral defects in large animal studies
Source: Cell Prolif. 2019 Sep 6;52(6):e12653. doi: 10.1111/cpr.12653 (PMC6869519; doi:10.1111/cpr.12653)
Supplement: Supplementary file 6 [file CPR-52-e12653-s006.docx]

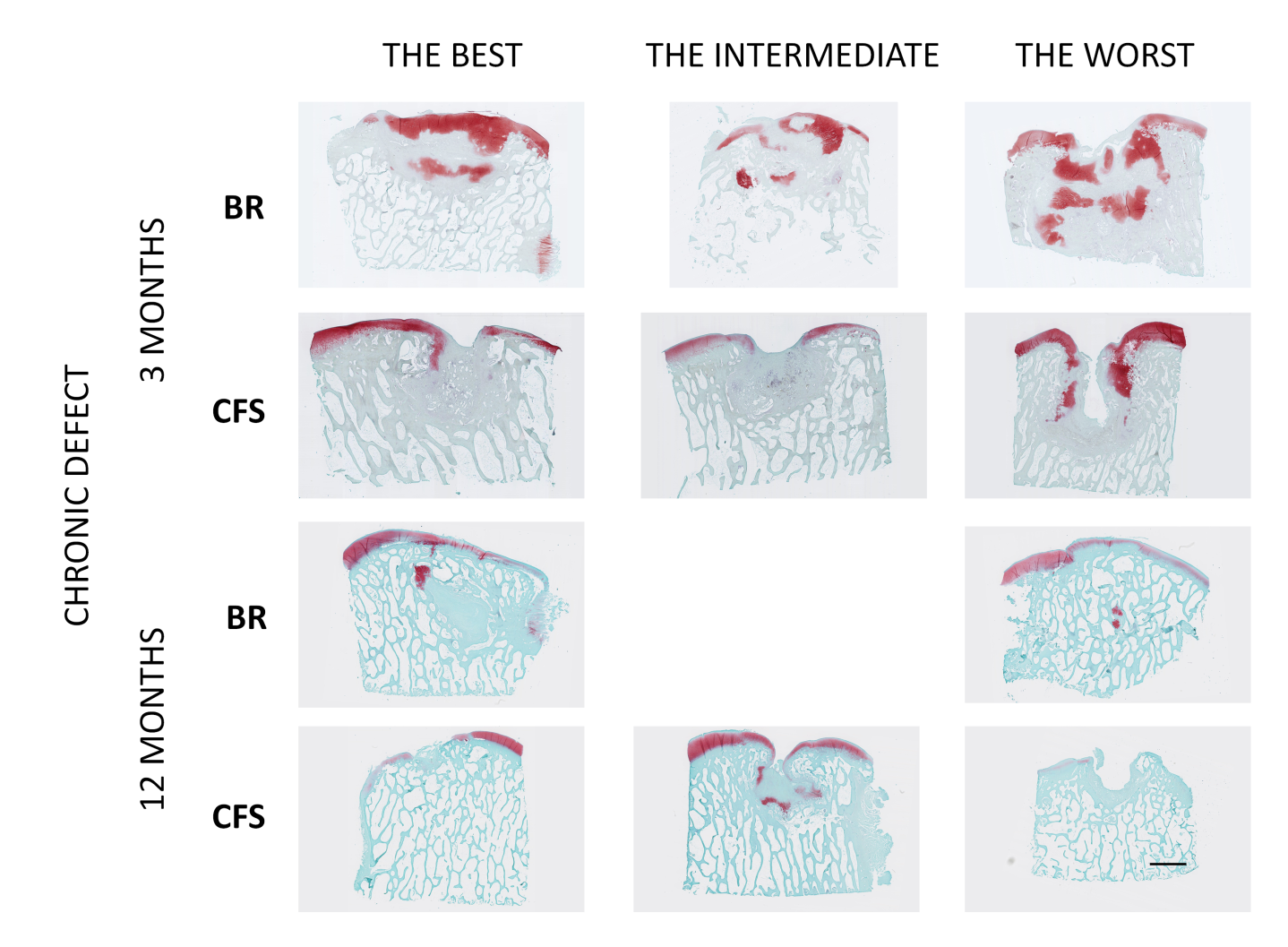


**Figure S6**. Safranin-O stained sections of the repaired osteochondral defects from the chronic study. Examples of the best, the intermediate and the worst result in each treatment group are provided.
